# Improved Solution-based SERS Detection of Creatinine by Inducing Hydrogen-Bonding Interaction for Effective Analyte Capture

**DOI:** 10.1016/j.talanta.2024.126373

**Published:** 2024-06-06

**Authors:** Supriya Atta, Tuan Vo-Dinh

**Affiliations:** a Fitzpatrick Institute for Photonics, Duke University, Durham, NC 27708, USA.; b Department of Biomedical Engineering, Duke University, Durham, NC 27708, USA.; c Department of Chemistry, Duke University, Durham, NC 27708, USA.

**Keywords:** Silver-coated gold nanostars, enrichment, SERS, MPA, creatinine, CKD

## Abstract

Recently, solution-based surface-enhanced Raman scattering (SERS) detection technique has been widely recognized due to its cost-effectiveness, simplicity, and ease of use. However, solution-based SERS is limited for practical applications mainly because of the weak adsorption affinity of the target biomolecules to the surface of plasmonic nanoparticles. Herein, we developed a highly sensitive solution-based SERS sensing platform based on mercaptopropionic acid (MPA)-capped silver-coated gold nanostars (SGNS@MPA), which allows efficient enrichment on the nanostars surface for improved detection of an analyte: creatinine, a potential biomarker of chronic kidney disease (CKD). The SGNS@MPA exhibited high enrichment ability towards creatinine molecules in alkaline medium (pH-9) through multiple hydrogen bonding interaction, which causes aggregation of the nanoparticles and enhances the SERS signal of creatinine. The detection limit for creatinine was achieved at 0.1 nM, with a limit of detection (LOD) value of 14.6 pM. As a proof-of-concept demonstration, we conducted the first quantitative detection of creatinine in noninvasive human fluids, such as saliva and sweat, under separation-free conditions. We achieved a detection limit of up to 1 nM for both saliva and sweat, with LOD values as low as 0.136 nM for saliva and 0.266 nM for sweat. Overall, our molecular enrichment strategy offers a new way to improve the solution-based SERS detection technique for real-world practical applications in point-of-care settings and low-resource settings.

## Introduction

Chronic kidney disease (CKD) has been a worldwide public health concern in recent decades, affecting approximately 10% of the world’s population. According to global estimates, over 750 million patients are affected annually by CKD-induced diseases, such as end-stage renal disease (ESRD), cardio-cerebrovascular disease, CKD-mineral bone disorder syndrome (CKD-MBD), dyslipidemia, diabetes, and anemia^1–4^. When the kidneys stop functioning properly, metabolic waste in the bloodstream, such as creatinine, accumulates in patients’ bodies. The creatinine level in human body fluids is an important indicator for the development of CKD-related diseases ^5–7^. Therefore, it is important to develop a rapid, simple, and sensitive method for routine clinical analysis of creatinine using portable detection systems to prevent CKD-related diseases. Currently, the most common clinical methods for measuring creatinine levels in body fluids are Jaffe’s reaction and enzymatical assays^8, 9^. However, they suffer from poor sensitivity and selectivity^10^. Other analytical techniques such as high-performance liquid chromatography (HPLC)^11^, ion mobility mass spectrometry (IM-MS)^12^, chemiluminescence ^13^, and electrophoresis^14^ have been utilized for sensitive and accurate detection of creatinine. Although most of these methods have good sensitivity, they require elaborate sample preparation and highly trained technicians to operate them, which reduces the possibility of cost-effective point-of-need screening for renal diagnosis, especially in remote and underdeveloped areas with insufficient clinical support and limited resources^15^.

In this contest, surface-enhanced Raman spectroscopy (SERS) has received increasing attention as an analytical technique for rapid screening of analytes with high sensitivity, portability, and simultaneous detection of multiple mixtures^16–21^. Recently, solution-based SERS has emerged as an analytical technique that offers several advantages such as low cost, flexibility, and portability, which improves the possibility of rapid on-site SERS detection^22–25^. For instance, over three decades, our laboratory has developed various SERS-active platforms for a wide variety of sensing applications^26–28^. The sensitivity of solution-based SERS is dependent on mainly two factors. Firstly, it depends on the morphology of the plasmonic noble metal nanoparticles, which can enhance the Raman signal of the analyte^16^. The ultra-sensitive SERS signal can be achieved in the presence of sharp edges, tips, and intermetallic junctions known as “hot spots”^29^. Secondly, the SERS enhancement depends on the affinity of the target molecules towards plasmonic metal nanoparticles^30^. Generally, plasmonic metal nanoparticles have a high affinity towards thiol functional group molecules. However, most of the biomolecules, including creatinine, don’t possess any thiol group; thus, they can hardly be absorbed on plasmonic nanoparticle surfaces.

To enrich the weak affinity molecules on the plasmonic metal surfaces, researchers have developed new strategies to fabricate the plasmonic active metal nanoparticles with capping agents which can enrich the target molecules via nonbonding interactions such as hydrogen bonding interaction and π-π stacking interaction^25, 31–36^. There are some reports for solution-based SERS detection of creatinine^37–39^. However, these methods required sample pre-treatment to enhance the SERS detection making them impractical for use in cost-effective detection systems. Thus, we believe that surface fabrication of SERS active nanoprobe with a specific capping agent can enhance the binding affinity and allowing the target creatinine molecules to enrich and get closer to the SERS hotspots resulting in sensitive SERS detection of creatinine.

In this study, we demonstrated a simple solution-based SERS detection of creatinine using mercaptopropionic acid (MPA) capped sharp and long spiked silver-coated gold nanostars (SGNS@MPA). MPA was realized to allow close contact with weak-affinity creatinine through hydrogen-bonding interaction at an alkaline pH (pH-9). Using this SGNS@MPA as a microplate-based SERS sensor, the spot-to-spot variation of the SERS intensity of 30 spots of creatinine was 5.8 %, and the limits of detection (LOD) of creatinine was achieved as low as 14.6 pM. Finally, to demonstrate the potential of our SERS platform for practical application, we have investigated its ability to detect creatinine in realistic conditions the quantitative detection of creatinine in human saliva and sweat under separation-free conditions, achieving clinically relevant levels of detection as low as 0.136 nM and 0.266 nM for saliva and sweat, respectively. Since the detection of creatinine by our SERS method falls well below the normal clinical range, this meets the requirement of detecting and analyzing creatinine in point-of-care settings and in low-resource settings.

### Experimental section

#### Materials and Characterization.

Chloroauric acid (HAuCl_4_), L-ascorbic acid, silver nitrate (AgNO_3_, 99.8%) hydrochloric acid (HCl), trisodium citrate (Na_3_C_6_H_5_O_7_), and creatinine were purchased from Sigma-Aldrich (USA). Pooled human saliva and sweat samples were purchased from Innovative Research (USA). The morphology of nanostars was characterized by analysis using FEI Tecnai G2 Twin transmission electron microscope, and HAADF STEM images and EDS maps were acquired using Aberration Corrected STEM-Thermo Fisher Titan 80–300 (USA, North Carolina). UV-vis spectra were recorded using a Shimadzu UV-3600i spectrometer with cuvettes of 1-cm path length at room temperature (USA). Milli-Q water has been utilized in this study.

#### Synthesis of SGNS and SGNS@MPA.

SGNS was synthesized following a previously reported method^17^. We first synthesized gold nanostars and then coated them with silver. The synthesis of gold nanostars was as follows. A solution of 200 μL of 1 N HCl was added to a solution containing 50 mL of 1 mM HAuCl_4_ and 500 μL of the citrate-capped gold seed solution. Then, 1 mL of 3 mM AgNO_3_ and 1 mL of 100 mM ascorbic acid were added to the solution, and the solution was stirred for 2 minutes. The nanostars solution was used for the silver coating process, where the nanostars solution was diluted with 40 mL of water; then, 3 mM ascorbic acid and 0.45 mM AgNO_3_ were added immediately to the nanostars solution. SGNS nanoparticles were further functionalized with 2 μM MPA solution and then stored at room temperature.

## Results and Discussion

**Figure 1.**
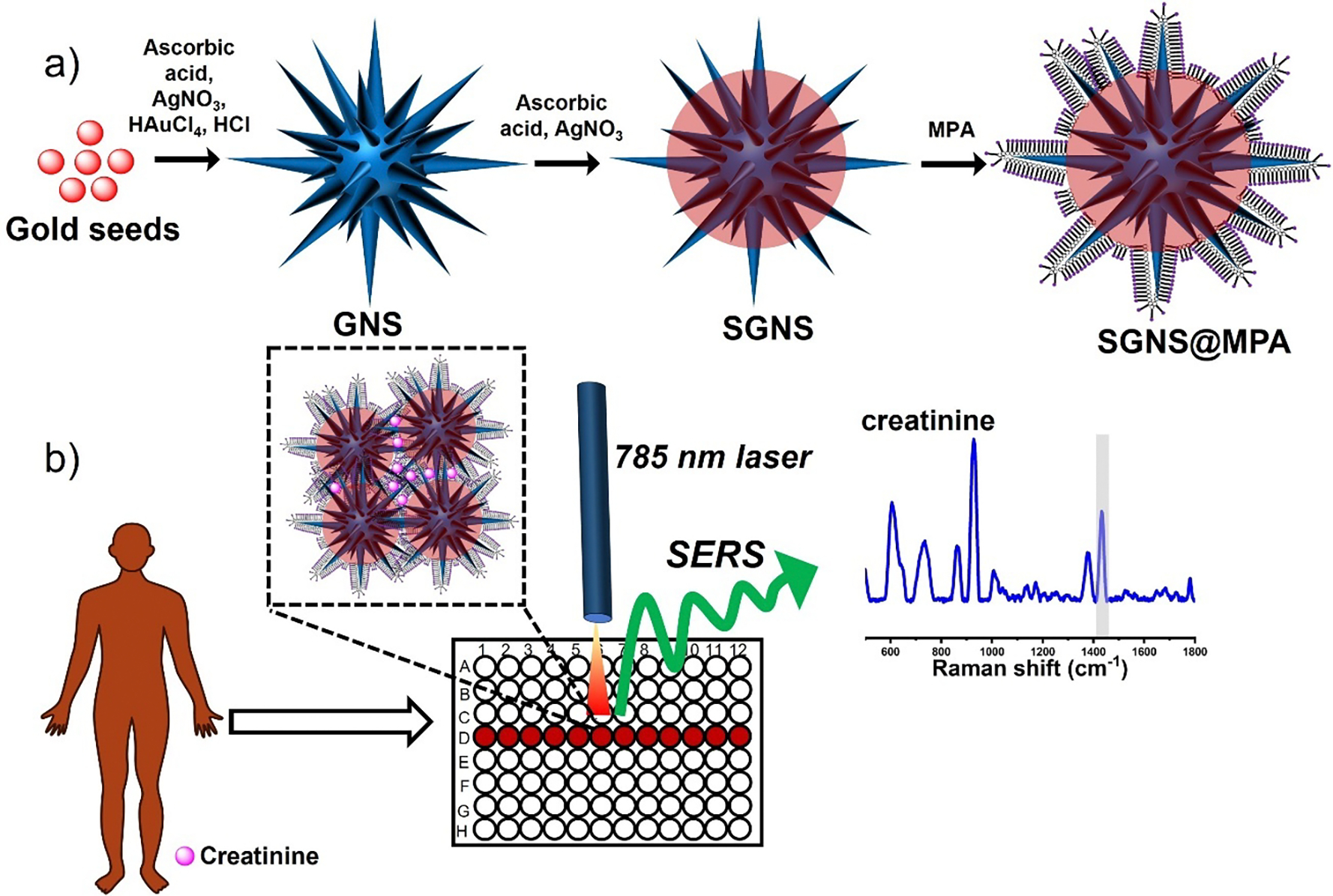
Schematic representation of the SGNS@MPA synthesis (a) and microplate-based SERS detection platform for creatinine detection in human saliva (b).

In this study, we have selected SGNS as a SERS probe because of its unique ultra-strong electromagnetic enhancement properties arising from the high scattering silver coating and multiple numbers of sharp spikes of GNS^17^. The schematic image in Figure 1 illustrates the synthesis of the SERS probe SGNS@MPA and a simple, easy-to-use solution-based SERS detection of creatinine in human fluids without any sample pre-processing steps. In the first step, we synthesized SGNS through a seed-mediated surfactant-free GNS synthesis method (Figure 1a). It is important to emphasize that the formation of spikes on GNS is crucial for achieving the maximum SERS effect^40^. To achieve maximum number of spikes on GNS, AgNO_3_ plays a crucial role in the growth of GNS spikes, acting as a growth factor in GNS synthesis^41^. The reduction and deposition of Ag^+^ on the seeds results in the creation of multiple twin facets, which are essential for spike formation. Additionally, it has been reported that Ag^+^ deposition on the spikes helps stabilize their morphology^40, 41^. SGNS was capped with a bifunctional small ligand MPA, an organosulfur compound containing two functional groups: a thiol group (-SH) and a carboxylic acid group (-COOH). The thiol group has a strong affinity to bind on the SGNS surface through Au/Ag-S bonding. On the other hand, the carboxylic acid group facilitates the nanoparticles to disperse well in polar solvents. We believe that the amine group of creatinine is protonated at an acidic pH (4.5) and anchors to SGNS@MPA^42–44^. Subsequently, they form strong hydrogen bonds at pH 9. Figure 1b shows the solution-based SERS detection of creatinine. To achieve ultra-strong SERS enhancement of creatinine, molecular enrichment of creatinine on the SGNS surface is necessary, which can be possible through strong hydrogen bonding interactions of MPA with creatinine. Due to the hydrogen bonding interaction between COOH of MPA and NH_2_ of creatinine, the interparticle interaction increases, resulting in aggregation of the nanoparticles. Thus, the SERS signal of the target molecule creatinine was significantly enhanced by generating hot spots at the nanometer gap.

**Figure 2.**
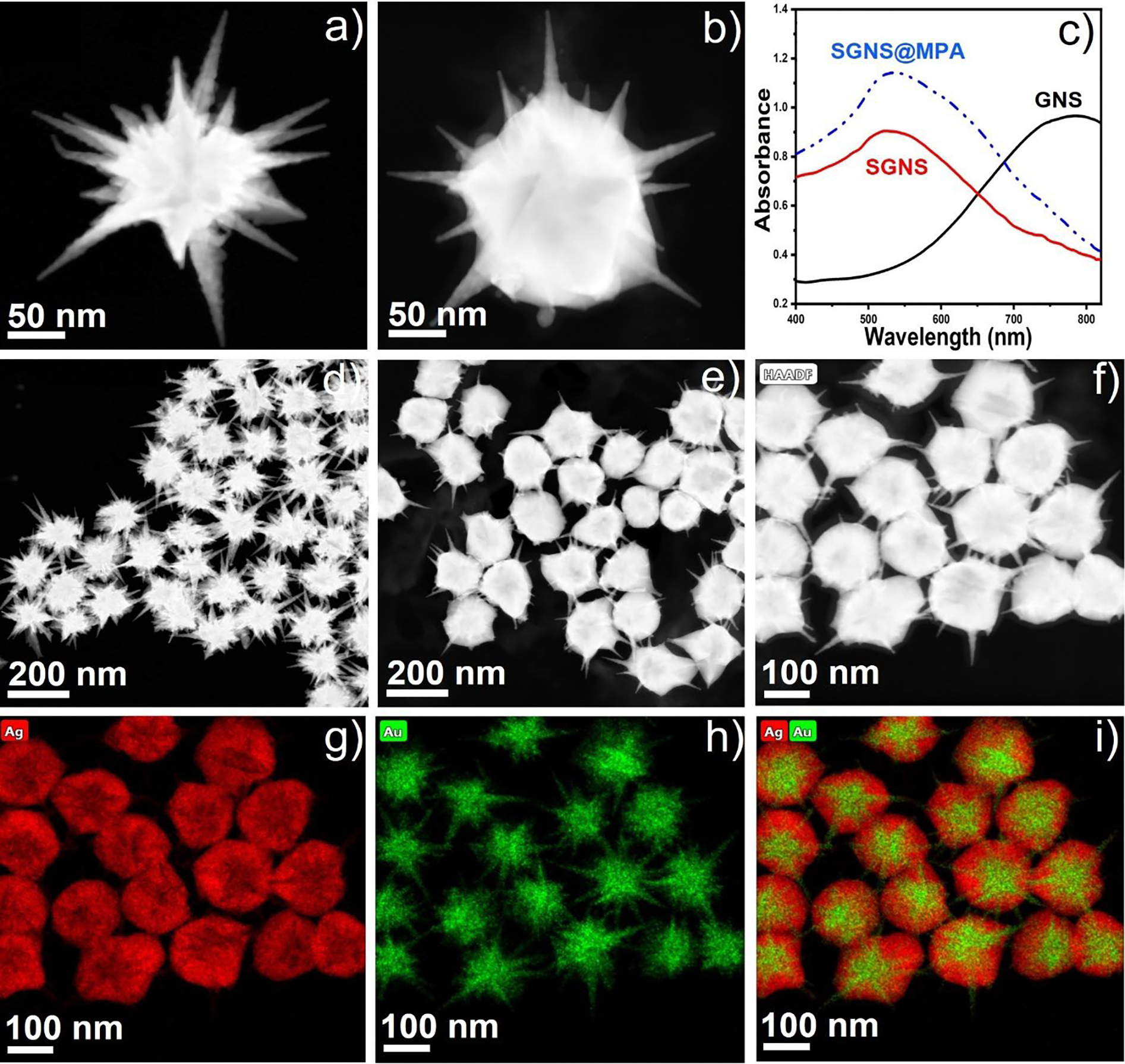
STEM images of GNS and SGNS (a-b). The UV-Vis absorbance spectra of GNS, SGNS, and SGNS@MPA (c) STEM images of multiple GNS and SGNS (d-e). The EDS mapping of multiple SGNS (f-i).

Figure 2a shows the representative scanning transmission electron microscopy (STEM) images of GNS, which indicates that the average spike length of the GNS was 90 ± 5 nm. Figure 2b shows the STEM image of SGNS, whose core diameter was increased from GNS. The UV-Vis absorbance spectra shows that the main localized surface plasmon resonance (LSPR) peak of GNS was blue-shifted from 790 nm to 520 nm (Figure 2c). The blue shift of the plasmon resonance peak of SGNS becomes evident as silver is deposited on the GNS^17^. Previous reports have shown that silver coating on gold nanostars causes a blue shift in the plasmon resonance peak, and this effect intensifies with increased silver coating on the GNS^17^. Interestingly, the plasmon band of SGNS was red shifted from 520 nm to 524 nm upon MPA addition. Figure 2d–e shows multiple numbers of GNS and SGNS, indicating that the synthesis of GNS and SGNS was uniform. The energy-dispersive X-ray (EDS) spectroscopic measurements of the SGNS morphology reveal that silver was selectively deposited at the core of GNS (Figure 2f–i).

**Figure 3.**
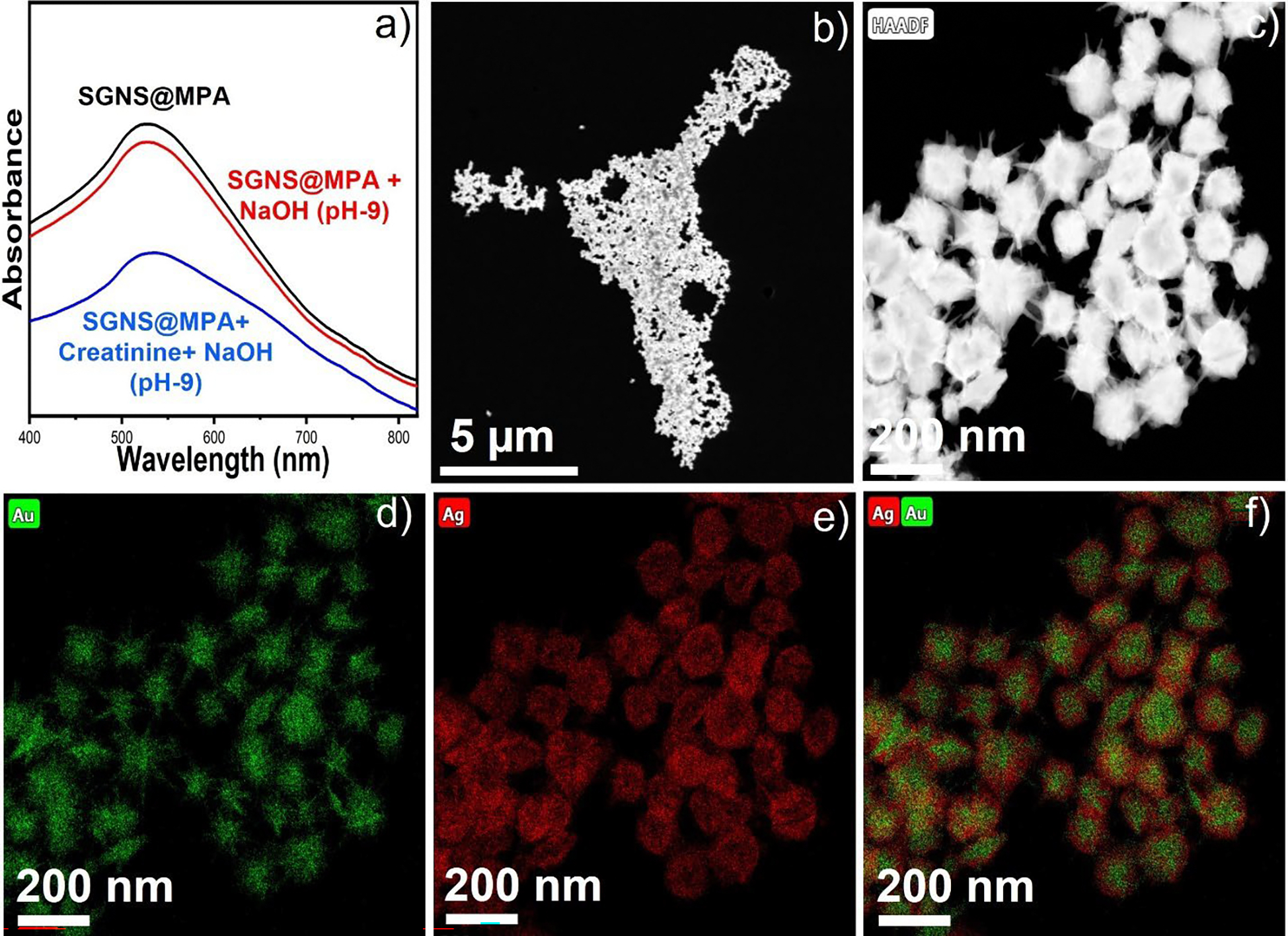
The UV-Vis absorbance spectra of SGNS@MPA and a mixture of SGNS@MPA and creatinine at pH-9 (a). STEM image of SGNS@MPA after addition of creatinine at pH-9 shows that the nanoparticles are close to each other and tend to aggregate (b). The EDS mapping of multiple SGNS shows the morphology of the SGNS@MPA was retained (c-f).

We measured the pH levels of SGNS and SGNS@MPA, which were found to be 4.6 and 4.5, respectively. The pH of SGNS was found to be acidic. As anticipated, the AgNO_3_ was reduced in the presence of ascorbic acid and deposited on the GNS, resulting in an acidic pH of the SGNS solution. It is important to note that an alkaline pH is necessary to deprotonate the acidic functional capping agents present on the nanoparticle’s surface and form strong hydrogen bonds with the amine group of creatinine. Indeed, SGNS possesses a weak capping ligand: ascorbic acid, which can capture the target creatinine molecules through hydrogen bonds. Unfortunately, ascorbic acid is a weak binding ligand and causes aggregation of nanoparticles in alkaline medium. The UV-vis absorbance spectra shows that the plasmon peak was reduced and broadened, indicating aggregation of the SGNS (Figure S1). We have performed a control experiment to investigate the stability of SGNS@MPA in an alkaline medium. Interestingly, the UV-Vis absorbance spectra exhibited that the plasmon peak was retained (Figure 3a), which indicates that MPA stabilizes the nanoparticles and NaOH doesn’t induce aggregation of nanoparticles.

We further investigated the stability of SGNS@MPA with creatinine in an alkaline medium at pH=9. Interestingly, the UV-vis absorbance spectra show that the plasmon peak of SNS@MPA was reduced and broadened, indicating aggregation of the SGNS@MPA (Figure 3a). The STEM image also confirmed that the nanoparticles were aggregated (Figure 3b). Interestingly, the STEM-EDS images revealed that the morphology of the SGNS was retained (Figure 3c–f). Collectively, this controlled study indicates that the presence of creatinine drives the aggregation of SGNS@MPA at pH=9, which might be due to multiple hydrogen bonding interactions and cross-linking of creatinine-inducing aggregation of nanoparticles^45, 46^.

**Figure 4.**
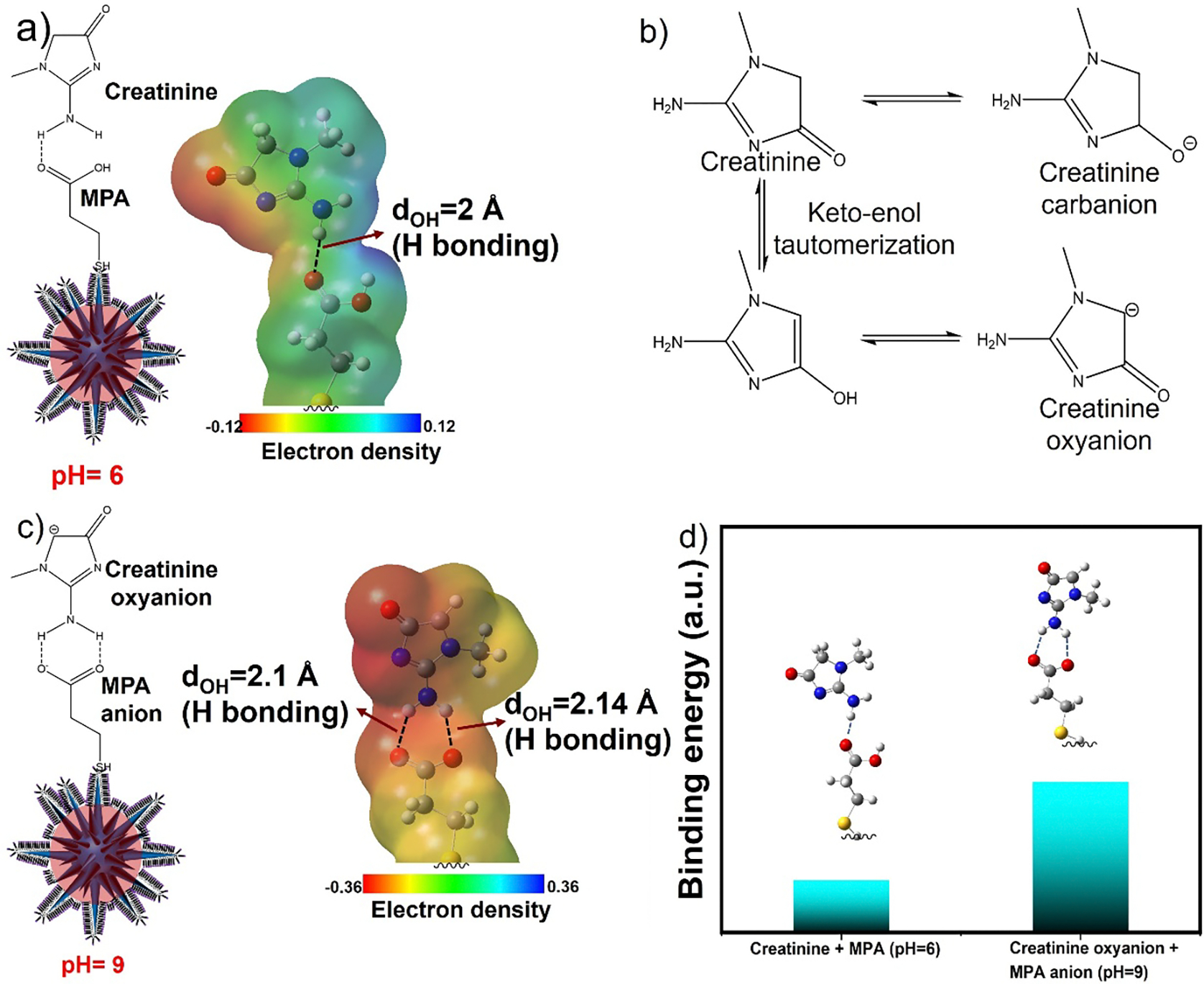
3D model and DFT simulation with electron density map indicate interactions (hydrogen bonding) between MPA and creatinine at pH-6 (a). Schematic representation of the tautomerism of creatinine and formation of carbanion and oxyanion (b). 3D model and DFT simulation with electron density map indicate interactions (hydrogen bonding) between MPA anion and creatinine oxyanion in alkaline condition (pH=9) (c). Relative binding energy comparison between MPA and creatinine in acidic (pH=6) and alkaline condition (pH=9) (d).

To investigate the molecular interaction of MPA and creatinine and the pH dependence aggregation of SGNS@MPA in the presence of creatinine, we performed density functional theory (DFT) calculations of MPA and creatinine in acidic and alkaline conditions. We first studied the hydrogen bonding interaction in acidic conditions where the carboxylic acid group (-COOH) of MPA and the amine group (-NH_2_) of creatinine were protonated. The DFT calculation shows only one hydrogen bonding interaction occurs between the amine group (-NH_2_) of creatinine and the carboxylic acid group (-COOH) of MPA. The distance between N-H and COOH bond is 2.1 Å, which is shorter than the sum of its individual van der Waals radii of H and O (1.63 Å), indicating non-covalent interaction between these atoms (Figure 4a). As shown in Figure 4b, creatinine has an amine group that can form a carbanion at an alkaline pH (pH-9) and it can tautomerize to creatinine oxyanion^45^. Interestingly, there occur two hydrogen bonding interactions between the amine group and the acid group of MPA in alkaline condition (Figure 4c). Moreover, the binding energy calculation shows that the bond energies of the hydrogen bonds are strong and comparable to the bond energy of the creatinine binding to the SGNS@MPA in acidic condition, representing a stable six-membered ring complexation of the creatinine anion with the carboxylate group of MPA, via two hydrogen bonding interactions (Figure 4d). The optimized DFT calculated results for creatinine and MPA interaction were described in the Supporting Information (see Optimized DFT calculation of creatinine and MPA in SI).

**Figure 5.**
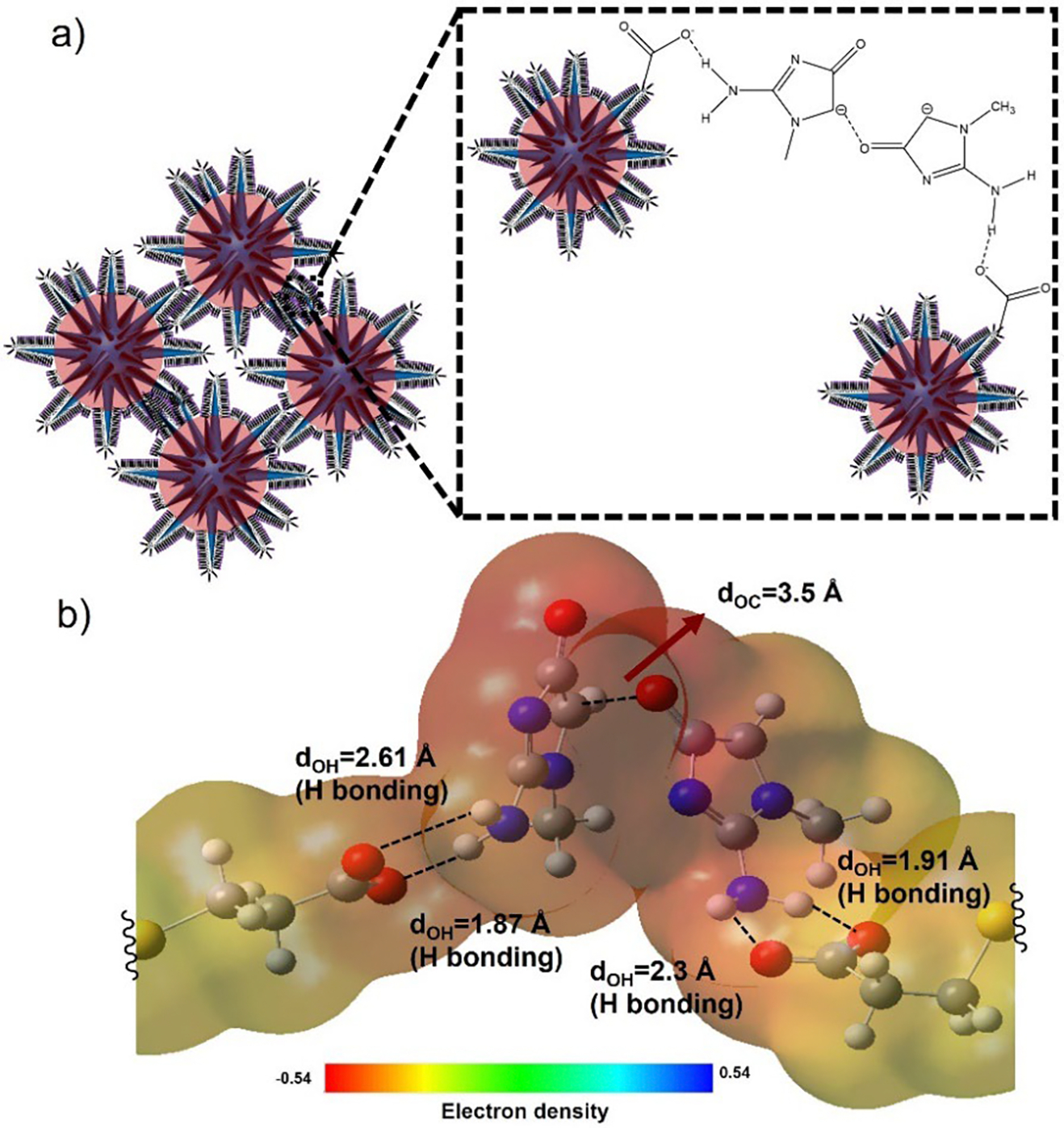
3D model of aggregated SGNS@MPA where two creatinine oxyanions were placed near two MPA anions (a) and DFT simulation with electron density map of multiple creatinine oxyanion and MPA anion interactions indicating multiple hydrogen bonding formations and cross-linking of creatinine oxyanions (b).

We further investigated the DFT calculation of the cross-linking interaction of creatinine, where two creatinine oxyanions were placed near two MPA anions (Figure 5a). The DFT calculation suggests weak non-bonding interaction occurs between the carboxyl group (C=O) of one oxyanion and the carbanion group of another oxyanion (Figure 5b). We believe that multiple cross-linking of creatinine occurs in the solution environment, which causes the aggregation of nanoparticles.

**Figure 6.**
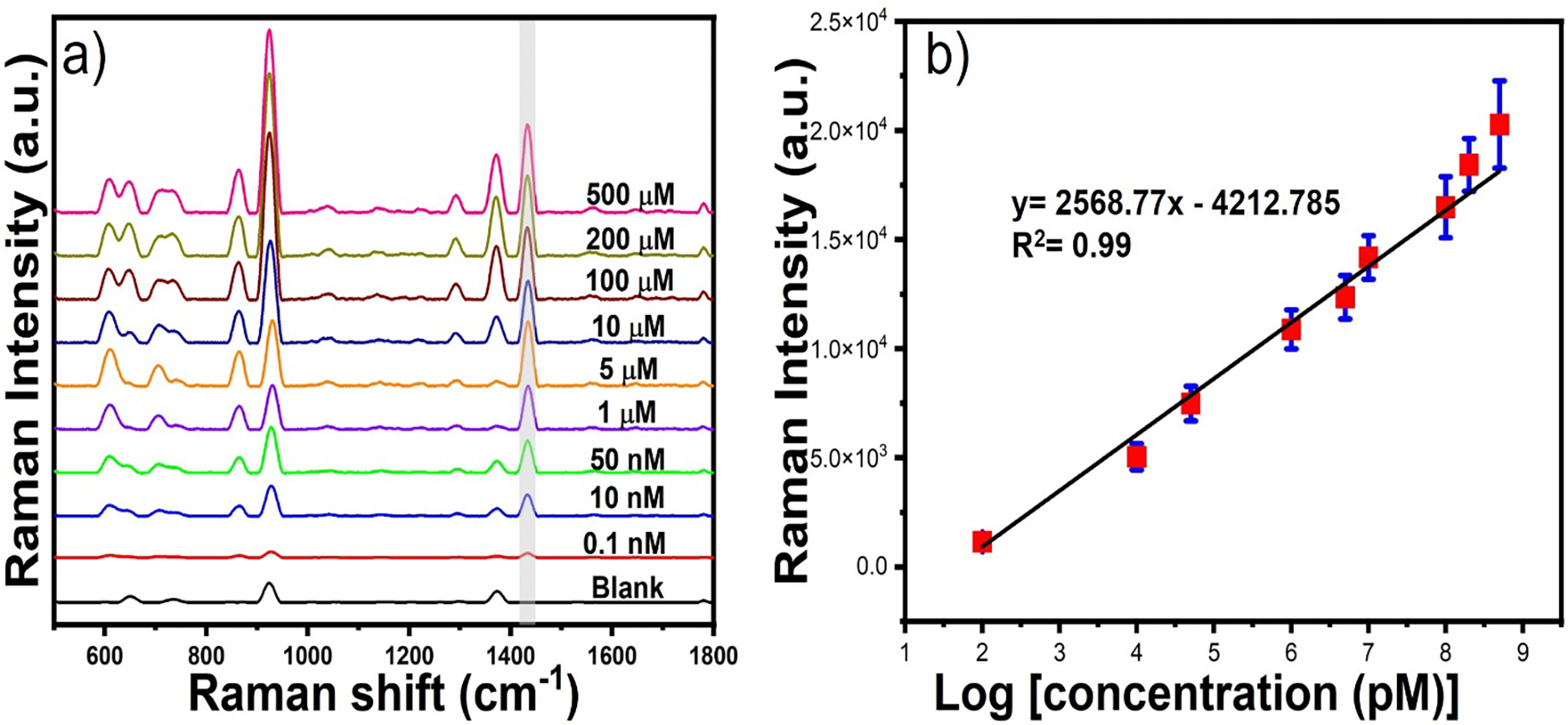
The SERS spectra and calibration curve of creatinine at different concentrations of creatinine ranging from 500 μM to 0.1 nM (a-b).

Next, we performed the solution-based SERS detection of creatinine, where a certain concentration of creatinine was added to the synthesized SGNS@MPA. We first added 1 mM creatinine to the SGNS@MPA solution. Interestingly, we observed that the SERS signal intensity of creatinine was very poor (Figure S2). As we discussed previously, the molecular interaction between creatinine and MPA was not strong enough in acidic condition to enrich the maximum amount of creatinine on the SGNS surface; thereby, the SERS signal was very poor. To enrich the molecular interaction between creatinine and MPA, we introduced NaOH to the SGNS@MPA, where 3 μL of 1 mM NaOH was added to the solution mixture so that the pH reached up to 9. We observed an immediate color change in the solution, indicating that the nanoparticles were aggregated, indicating the formation of strong hydrogen bonding, and cross-linking of creatinine. As depicted in Figure 6a, the characteristic SERS peaks of creatinine were observed at 872 cm^−1^, 925 cm^−1^, and 1438 cm^−1^ which are attributed to the C=O stretching, C-C stretching and CH_3_ anisometric deformation, respectively^47^. The SERS signal intensity was determined for a range of creatinine concentration from 500 μM to 0.1 nM. The SERS intensity of creatinine at 1438 cm^−1^ was used to establish the calibration curve. A linear correlation was established between the SERS intensity of creatinine and logarithmic value of the concentration of creatinine that can be fitted with the straight-line y= 2568.77 log C_creatinine_ (pM) - 4212.785 (R^2^ = 0.99) (Figure 6b). The detection limit for creatinine was established at 0.1 nM, with a limit of detection (LOD) of 14.6 pM. The LOD and LOQ were estimated using a standard equation according to the IUPAC definition: LOD = 3σ/S, and LOQ=10σ/S, where σ and S represent the standard deviation of blank measurements and the slope of the linear equation, respectively. The LOD and LOQ were determined to be as low as 14.6 and 48.62 pM. The extremely low LOD of creatinine using our simple solution-based SERS platform shows an advantage over other methods previously reported in the literature^37, 47^. We further investigated the spot-to-spot variation of the SERS intensity of 30 spots of creatinine. Figure S3 shows the SERS peak intensity of creatinine at 10 μM concentration for 30 different samples where the RSD value was 5.8 % indicating good reproducibility of our solution-based SERS platform.

To validate the specificity of our SERS platform, we performed the SERS measurements of several interfering agents including uric acid, albumin, urea, and glucose at a concentration of 10 μg/mL. As depicted in Figure S4, the SERS spectra show notable selectivity for the characteristic Raman peak of creatinine at 1438 cm^−1^, distinguishing it from other interfering small molecules and proteins.

**Figure 7.**
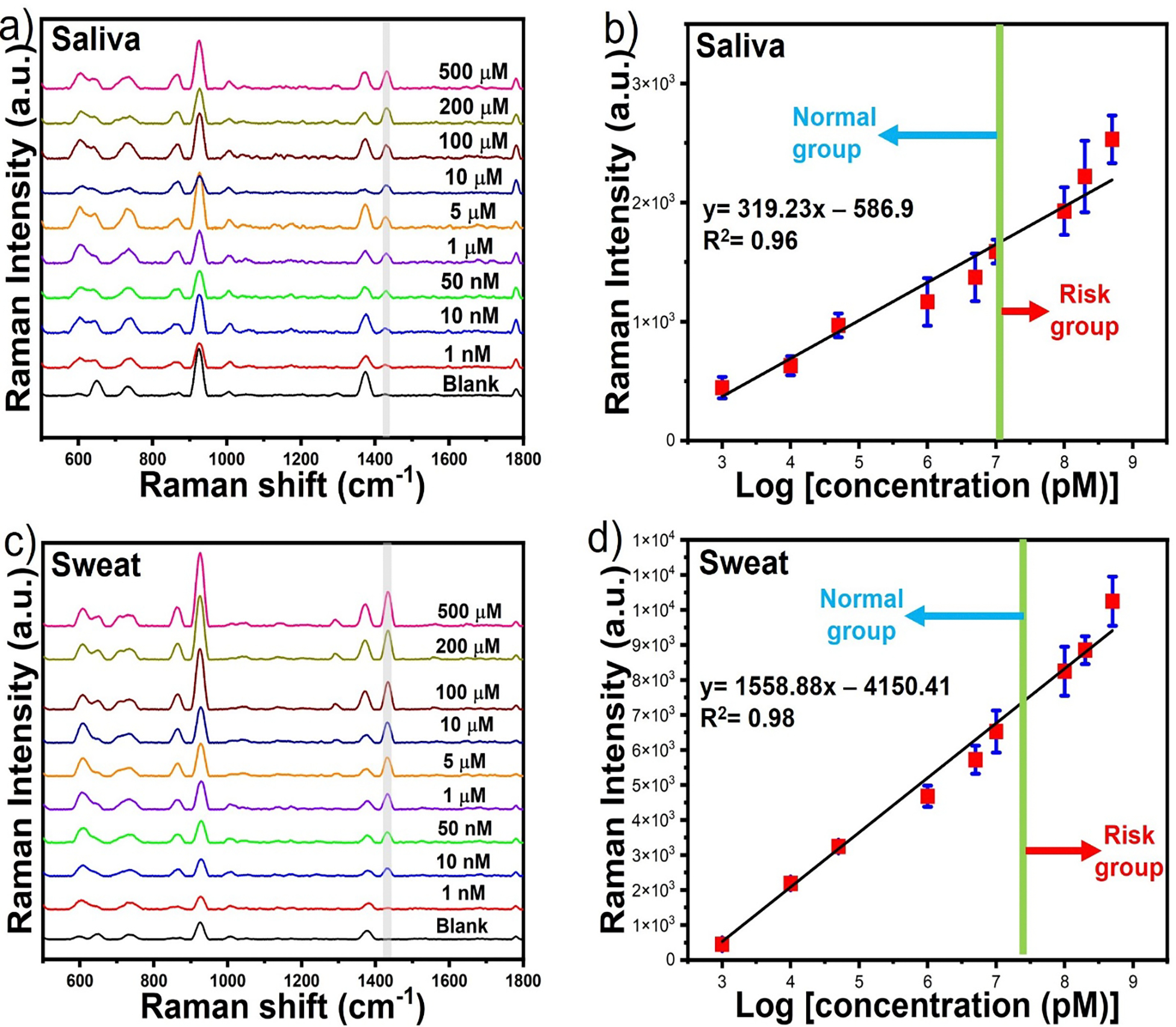
The SERS spectra and calibration curve of creatinine spiked in human saliva at different concentrations of creatinine ranging from 500 μM to 1 nM (a-b). The SERS spectra and calibration curve of creatinine spiked in human sweat at different concentrations of creatinine ranging from 500 μM to 1 nM (a-b).

To illustrate the potential of the SERS platform for use as a rapid point-of-need diagnostic system, we have performed quantitative detection of creatinine in easily accessible human body fluids such as human saliva and sweat under separation-free conditions. Saliva and sweat were selected as noninvasive human fluids for creatinine detection. A range of creatinine concentration from 500 μM to 1 nM was spiked in human saliva samples (Figure 7a). The characteristic SERS peak at 1438 cm^−1^ was used to plot the calibration curve (Figure 7b). A linear correlation was established between the SERS intensity of creatinine and logarithmic value of the concentration of creatinine that can be fitted with the straight-line for the saliva samples, where the linear equations for saliva was y= 319.23 log C_creatinine_ (pM) – 586.9 (R^2^= 0.96). A similar set of experiments was performed for creatinine spiked in human sweat samples (Figure 7c). A linear correlation was plotted between the SERS intensity of creatinine and the logarithmic value of the concentration of creatinine (Figure 7d). The linear correlation was y= 1558.88 log C_creatinine_ (pM) – 4150.41 (R^2^= 0.98). The quantitative detection of creatinine reached a detection limit of up to 1 nM for both saliva and sweat, with LOD values as low as 0.136 nM for saliva and 0.266 nM for sweat. The LOQ was determined to be as low as 0.45 nM and 0.89 nM for saliva and sweat, respectively. The clinical range of creatinine concentration in saliva and sweat was around ~ 1.5 μM ^48^, and ~ 30 μM ^49^, respectively, indicating that our proposed solution-based SERS detection platform can effectively distinguish between normal groups and groups at risk. Overall, our SERS assay protocol is suitable for cost-effective and rapid SERS detection of creatinine in real-life samples as the analytes can be delivered directly to the gold nanoparticle solution for subsequent detection without the need for sample treatment. In this context, to the best of our knowledge, SGNS@MPA provides a practical platform that yields the highest sensitivity for creatinine detection where most of the reported methods required time-consuming SERS substrate preparation or sample repurification to achieve good SERS sensitivity of creatinine^39, 50, 51^. Lastly, our SERS measurement takes only a second, making it suitable for rapid on-site screening applications for early kidney diagnosis in low-resource settings. Based on the findings of this study, the proposed system has the potential to be used for liquid biopsy in the future.

We believe this is the first report demonstrating the detection of creatinine under separation-free conditions using solution-based SERS detection methods. Our SERS measurement results for creatinine detection, compared with other reported SERS-sensing approaches (Table 1), show significantly higher sensitivity for detecting creatinine in biological samples without any sample preprocessing. Notably, other reported solution-based SERS detection methods for creatinine require sample pre-treatment to enhance detection, making them impractical for rapid, point-of-care use^39^. Similarly, some reports on solid substrate-based SERS detection of creatinine involve cost-intensive substrate preparation, limiting their practicality for cost-effective detection systems^47, 50^. In contrast, our SERS detection method eliminates the need for additional pre-treatment steps, enhancing its applicability for rapid, on-site detection of creatinine, which could be beneficial in low-resource settings. Overall, we demonstrate the significant advantages of using small molecules like MPA for functionalization on highly SERS-active SGNS sensors to accurately quantify the biomarker creatinine. We believe our strategy could be applied to functionalize small molecules on SGNS sensors to enrich and detect other biomarkers of interest.

## Conclusion

In summary, we presented a rapid sensitive solution-based SERS platform integrated with SGNS@MPA for the detection of creatinine. The capping agent MPA facilitated enriching the weak affinity creatinine molecules on the SGNS surface and caused aggregation of nanoparticles, which increases the SERS sensitivity of creatinine. We further investigated the molecular interaction between MPA and creatinine and the pH dependence of SGNS@MPA aggregation using theoretical DFT calculations, which are in agreement with the experimental aggregation of nanoparticles in the presence of creatinine at pH=9. The SERS detection of creatinine exhibits trace quantification of creatinine with LOD achieved up to 14.6 pM. In addition, as proof of concept, we studied for the first time the quantitative detection of creatinine spiked in human body fluids such as human saliva and sweat under separation-free conditions, achieving clinically relevant levels of detection down to 0.136 nM and 0.266 nM for saliva and sweat, respectively. Overall, the performance of the SERS sensor SGNS@MPA makes it suitable for the diagnosis of creatinine in clinics in low-resource areas.

## Supplementary Material

Supplementary Material

## Figures and Tables

**Table 1.** Literature survey on the analytical performance of various SERS substrates for creatinine detection.

SERS substrate	LOD	Ref.

Au nanocubes	11 nM (water)	^37^
Au nanoparticles	12.8 mM	^39^
PAA/PAH bilayer coated Au film	1.47 μM	^52^
Au nanoparticles on blu-ray DVD (BRDVD)	0.28 μM	^47^
Self-assembled nano-Ag/Au@Au film	5 μM	^53^
Nanoporous gold disk	13.2 nM	^54^
Au nanoparticles	13.2 nM (water), 60 μM (urine)	^55^
**SGNS@MPA**	**0.136 nM (saliva)** **0.266 nM (sweat)**	**This work**
