# Supplementary material for: Improved Solution-based SERS Detection of Creatinine by Inducing Hydrogen-Bonding Interaction for Effective Analyte Capture
Source: Talanta. Author manuscript; Available in PMC 2025 Jun 5. (PMC12140409; doi:10.1016/j.talanta.2024.126373)
Supplement: Supplementary Material [file NIHMS2080249-supplement-Supplementary_Material.docx]

**Supporting Information**

**Improved Solution-based SERS Detection of Creatinine by Inducing Hydrogen-Bonding Interaction for Effective Analyte Capture**

*Supriya Atta, ^a, b^ Tuan Vo-Dinh ^a,^ ^b, c‡^*

^a^ Fitzpatrick Institute for Photonics, ^b^ Department of Biomedical Engineering, ^c^ Department of Chemistry, Duke University, Durham, NC 27708, USA.

‡ Correspondence author: [tuan.vodinh@duke.edu](mailto:tuan.vodinh@duke.edu)

**Theoretical simulation and Raman measurements**

**Density Functional Theory (DFT) Calculations.** The DFT calculations were used to calculate the hydrogen bonding interaction energy of the amine group (-NH_2_) of creatinine and the carboxylic acid (-COOH) group of MPA. We have used the unrestricted B3LYP exchange-correlation functional tool of the gaussian computational chemistry package (GAUSSIAN 16, GaussView 6.1). The 6-31G (d, p) basis set was employed for all atoms except Ag. The LANL2DZ basis set was used for Ag where 6 Ag atoms cluster was optimized according to the reported procedure^1, 2^. MPA molecule was optimized using unrestricted B3LYP exchange-correlation functional tool and then placed at the tip of the triangular 6 Ag atoms. After geometry optimization of the 6 Ag atom cluster with MPA, creatinine was placed near the MPA (~3 Å), and the entire complex system was optimized. The binding energy is calculated by:

E_Binding energy_ = (E(B3LYP) _MPA_ + E(B3LYP) _Creatinine_) - (E(B3LYP) _MPA_-_Creatinine complex_)

**Raman Measurements.** The SERS measurement was carried out using a lab-built portable system having a 785-nm laser source (Rigaku Xantus TM-1 handheld Raman device), a fiber optic probe (InPhotonics RamanProbe), a spectrometer (Princeton Instruments Acton LS 785), and a CCD camera (Princeton Instruments PIXIS: 100BR-eXcelon). The laser power of the Rigaku Xantus TM-1 was set at 150 mW, and the CCD camera exposure time was set at 1s.

**SERS Measurements.** The SERS measurement involves a minimal sample preparation in which 294 μL of the SGNS@MPA were thoroughly mixed with 3 μL of analyte solution and 3 μL of 1 mM NaOH solution in a 96-well plate whose bottom was covered with aluminum foil to prevent signal interference from the polypropylene well plate.


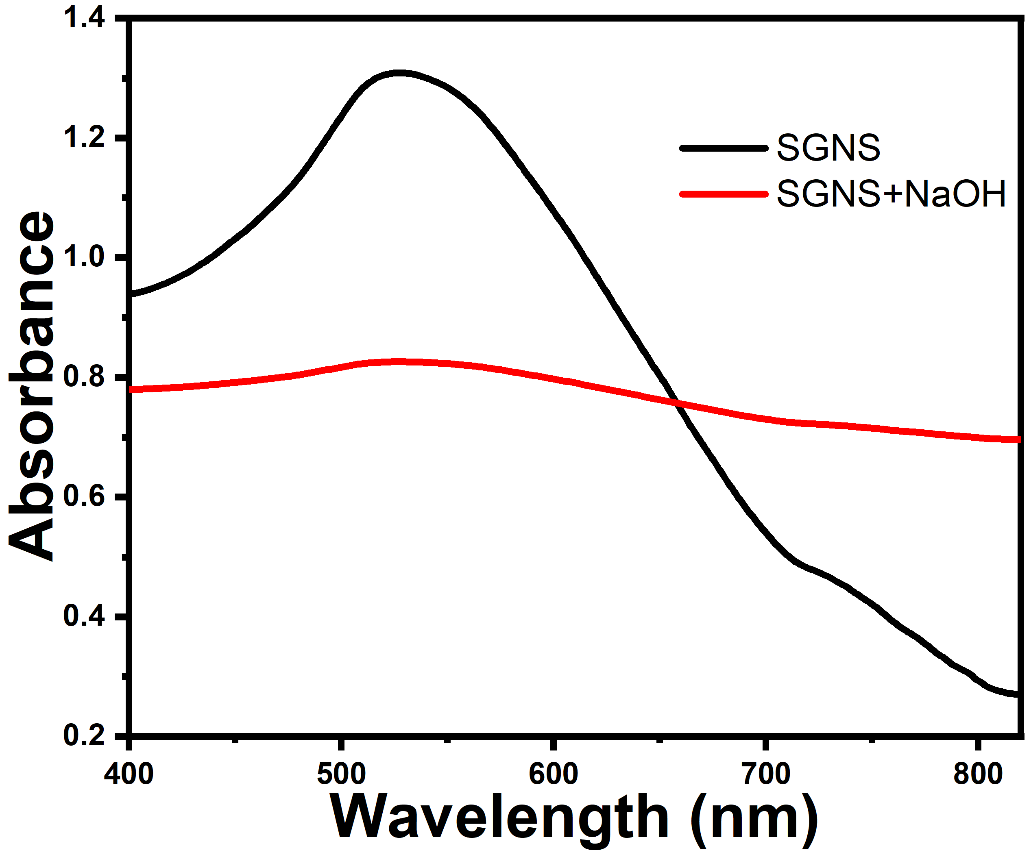


**Figure S1.** UV-Vis absorbance spectra of SGNS (black), and SGNS after NaOH addition (red).


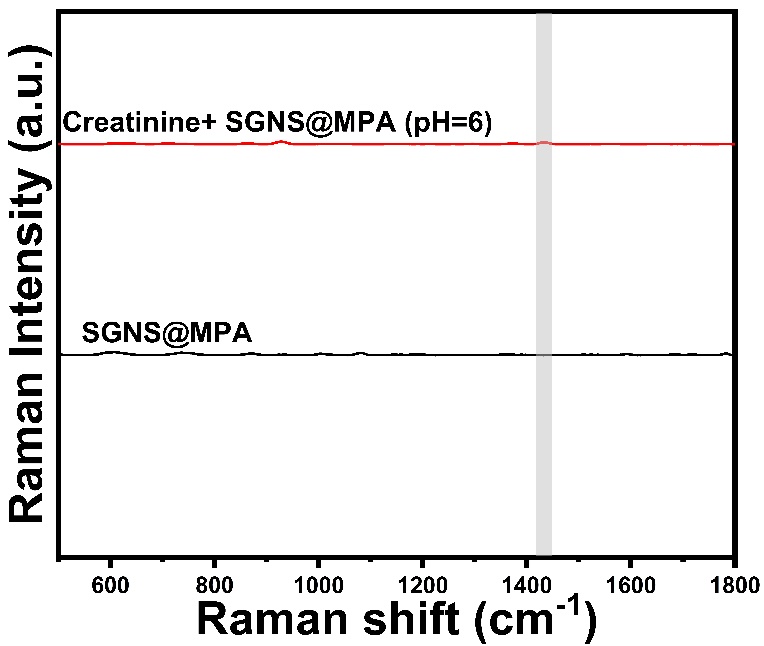


**Figure S2.** SERS spectra of SGNS@MPA (black), and after addition of creatinine at pH=6 (red).


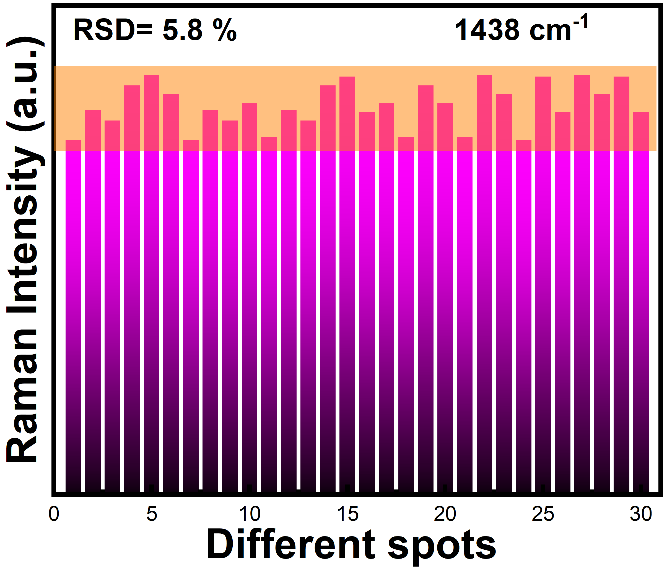


**Figure S3.** SERS peak intensity of creatinine at 1438 cm^-1^ for 30 different samples.


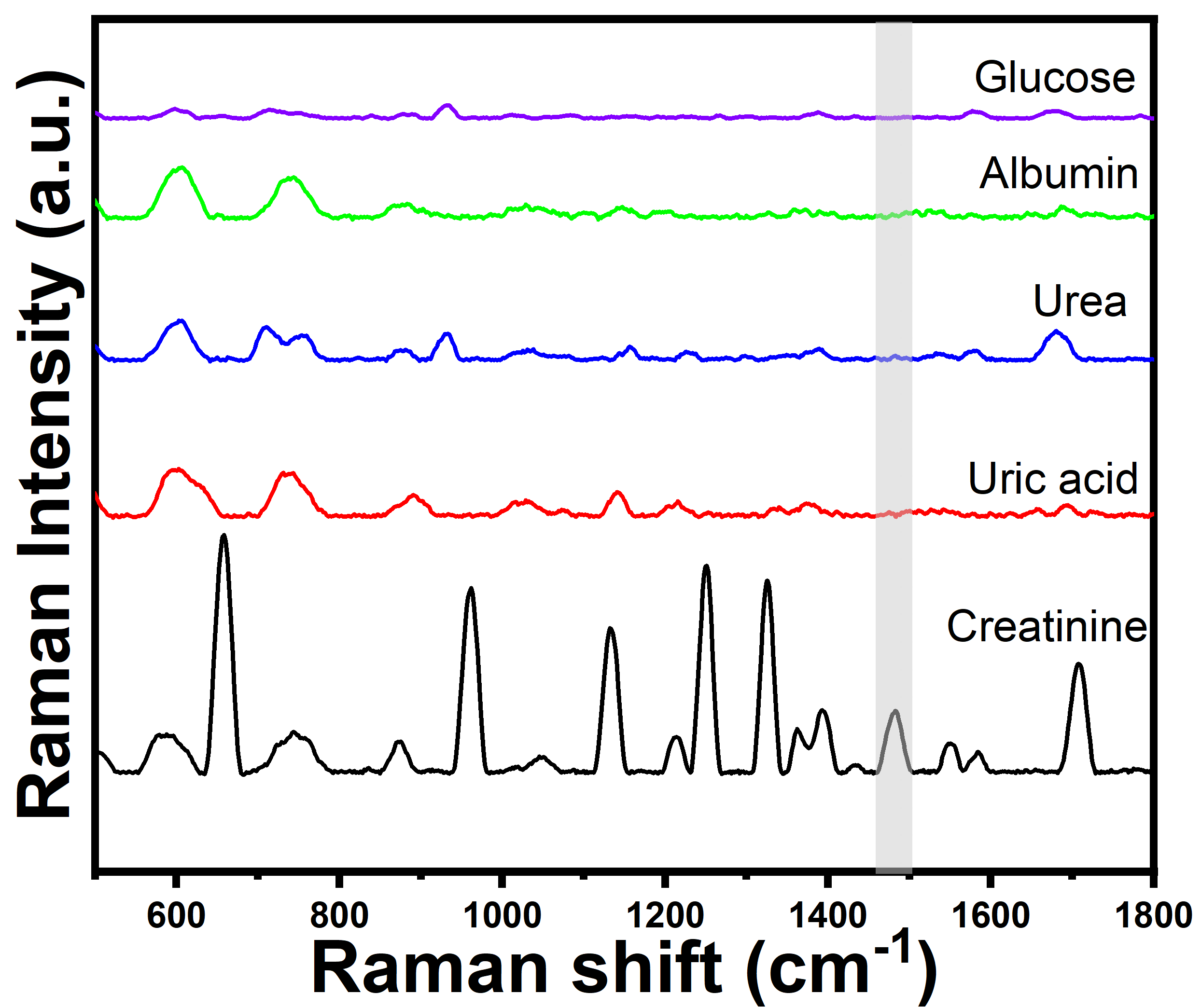


**Figure S4.** SERS spectra of creatinine, uric acid, urea, albumin, and glucose at 10 µM concentration.

**Optimized DFT calculation of creatinine and MPA-**

**Creatinine and MPA**

C 3.87396000 0.35990900 0.46341800

H 3.78865200 0.10593000 1.50770400

H 3.30852100 1.24714900 0.24553300

C 3.40457300 -0.79435700 -0.42394700

H 3.55067600 -0.52766800 -1.46502100

H 3.94799900 -1.70223300 -0.21385000

S 5.70271800 0.69235000 0.08811900

C 1.93625300 -1.06233700 -0.21308300

O 1.44285100 -2.15600900 -0.06350900

O 1.21079600 0.06508600 -0.22937800

C -4.69791000 -0.20330000 0.12367200

C -4.01308700 1.21781300 -0.07738800

H -4.50549400 2.16081800 -0.17917700

N -3.68458600 -1.07973500 0.19821200

N -1.29970200 -0.79976600 0.07672600

H -1.07740500 -1.77849100 0.17949400

H 0.23769900 -0.12630100 -0.10873100

O -5.89580000 -0.30603900 0.17815300

C -2.50688000 -0.48170100 0.08215500

N -2.76705800 1.03846500 -0.09683600

C -1.66278600 2.00115100 -0.25265600

H -2.06957900 2.98939300 -0.40118800

H -1.05288500 1.96859000 0.63600700

H -1.06377400 1.70194600 -1.09735300

Ag 9.63855234 -0.96528548 1.69718957

Ag 12.28517534 -1.77014248 1.68113857

Ag 7.03563934 -0.03746848 1.67976157

Ag 11.79396734 0.94995752 1.68314257

Ag 9.04883034 1.86166852 1.68598057

Ag 11.16575134 3.64179352 1.68710457

**Creatinine anion-MPA-**

Ag -9.55424564 0.89838913 -2.51227582

Ag -6.90762264 0.09353213 -2.52832682

Ag -12.15715864 1.82620613 -2.52970382

Ag -7.39883064 2.81363213 -2.52632282

Ag -10.14396764 3.72534313 -2.52348482

Ag -8.02704664 5.50546813 -2.52236082

C -4.14990700 0.46558700 -0.29953800

H -4.17216000 1.29230900 0.41251700

H -3.71243700 0.82761200 -1.23029500

C -3.34045800 -0.70122900 0.25254100

H -3.78223000 -1.07525600 1.18383800

H -3.35694800 -1.54430000 -0.45049500

S -5.89472900 -0.07815000 -0.62850900

C -1.85045200 -0.34947900 0.53217100

O -1.44819800 0.78701000 0.15688600

O -1.18555100 -1.25626300 1.10482700

C 4.38258800 0.42581500 -0.68389900

H 5.10825500 1.00473600 -1.23698800

N 1.31435400 0.32134500 1.14299300

H 0.63289000 0.89704600 0.64226200

H 0.78168100 -0.53779800 1.29358500

N 2.99346800 -1.15210300 0.20634100

C 2.47827700 0.05052100 0.40870900

N 3.25027300 1.04991000 -0.09710800

C 3.03886900 2.47113500 0.03394400

H 2.25262200 2.65824300 0.76839700

H 3.95700400 2.96191400 0.37973900

H 2.74517600 2.94076300 -0.91544800

C 4.22279700 -0.95659100 -0.50651100

O 4.96298700 -1.93561000 -0.87262400

**Two creatinine oxyanion and two MPA**

C -2.25069194 0.95366065 1.68073837

C -0.88941294 2.69649365 1.33360037

H -0.09750694 3.25834165 0.83766937

N -2.48375894 1.76909265 2.71163237

N -2.83786894 -0.31148935 1.47276037

H -3.60217394 -0.42012935 2.14542437

H -3.31648994 -0.39746235 0.54594437

N -1.28634594 1.42709665 0.83338837

C -0.85764094 0.81986765 -0.40929563

H 0.20513706 1.05623465 -0.60263663

H -0.97765694 -0.27365135 -0.33805663

H -1.44799594 1.16853465 -1.28214763

C -1.61130794 2.90772165 2.53193337

O -1.57999694 3.88887165 3.36687637

C -8.26078100 -1.36265800 -0.45382800

H -8.19624200 -2.18434200 0.27318600

H -8.51143600 -0.44117300 0.09265900

C -6.93485400 -1.21051800 -1.19079700

H -6.72466300 -2.11379300 -1.79390200

H -6.97228800 -0.36970700 -1.90592100

S -9.61653700 -1.72198000 -1.67123300

C -5.72930500 -0.98809700 -0.22370000

O -4.62836500 -0.69536400 -0.79967700

O -5.94431900 -1.13024600 1.01620900

C 4.05153700 3.55448700 -1.38926600

H 4.70929500 4.41968600 -1.32249700

C 2.73477300 3.33319000 -0.92320400

O 1.88613500 4.15374400 -0.40729500

N 2.44803500 1.93453800 -1.11920600

C 3.54714500 1.38747300 -1.64437400

N 3.68649700 0.05546000 -2.07217600

H 4.47536100 -0.44857800 -1.61220100

H 2.88386800 -0.48850100 -1.73299300

N 4.54994800 2.30138200 -1.83020100

C 5.82945600 2.06629700 -2.45870200

H 5.81375600 1.08453500 -2.95577200

H 6.03146500 2.84144000 -3.22016300

H 6.66767700 2.07797600 -1.73506700

C 6.13345261 -4.24247123 0.94872812

H 6.32285761 -5.14432523 1.55024712

H 6.64899561 -4.35266423 -0.01691188

C 4.64186261 -4.01978823 0.72738212

H 4.21551061 -4.91416523 0.23457012

H 4.11636561 -3.92617523 1.69512912

C 4.29183861 -2.78246023 -0.14533788

O 3.06778761 -2.62050423 -0.42559288

O 5.27765961 -2.04278623 -0.49589188

S 6.93181261 -2.81242223 1.82876412

Ag -10.94253883 -7.63639842 -3.88634871

Ag -9.93875274 -6.80519932 -6.32646904

Ag -11.95263323 -8.33755348 -1.41159718

Ag -9.98484960 -4.94558522 -4.28197795

Ag -11.03440438 -5.74350450 -1.70733978

Ag -10.08571726 -3.18409968 -2.15415125

Ag 12.16270189 -2.64258904 5.29070208

Ag 10.96495516 -4.01755176 7.37097754

Ag 13.23650330 -1.23489639 3.16899141

Ag 9.34550980 -3.25650608 5.26421711

Ag 10.53002819 -1.80333618 3.06144260

Ag 7.83838505 -2.43899296 3.09606135

**References**

1. Nguyen, L. B. T.; Leong, Y. X.; Koh, C. S. L.; Leong, S. X.; Boong, S. K.; Sim, H. Y. F.; Phan-Quang, G. C.; Phang, I. Y.; Ling, X. Y., Inducing Ring Complexation for Efficient Capture and Detection of Small Gaseous Molecules Using SERS for Environmental Surveillance. *Angewandte Chemie International Edition* **2022,** *61* (33), e202207447.

2. You, T.-t.; Yin, P.-g.; Jiang, L.; Lang, X.-f.; Guo, L.; Yang, S.-h., In situ identification of the adsorption of 4,4′-thiobisbenzenethiol on silver nanoparticles surface: a combined investigation of surface-enhanced Raman scattering and density functional theory study. *Physical Chemistry Chemical Physics* **2012,** *14* (19), 6817-6825.
